# Parkinson’s disease with mild cognitive impairment may has a lower risk of cognitive decline after subthalamic nucleus deep brain stimulation: A retrospective cohort study

**DOI:** 10.3389/fnhum.2022.943472

**Published:** 2022-09-06

**Authors:** Hutao Xie, Quan Zhang, Yin Jiang, Yutong Bai, Jianguo Zhang

**Affiliations:** ^1^Department of Neurosurgery, Beijing Tiantan Hospital, Capital Medical University, Beijing, China; ^2^Beijing Key Laboratory of Neurostimulation, Beijing, China; ^3^Department of Functional Neurosurgery, Beijing Neurosurgical Institute, Capital Medical University, Beijing, China

**Keywords:** Parkinson’s disease, subthalamic nucleus, deep brain stimulation, cognitive decline, mild cognitive impairment, Montreal cognitive assessment

## Abstract

**Background:**

The cognitive outcomes induced by subthalamic nucleus deep brain stimulation (STN-DBS) remain unclear, especially in PD patients with mild cognitive impairment (MCI). This study explored the cognitive effects of STN-DBS in PD patients with MCI.

**Methods:**

This was a retrospective cohort study that included 126 PD patients who underwent STN-DBS; all patients completed cognitive and motor assessments before and at least 6 months after surgery. Cognitive changes were mainly evaluated by the Montreal cognitive assessment (MoCA) scale and the seven specific MoCA domains, including visuospatial/executive function, naming, attention, language, abstract, delayed recall, and orientation. Motor improvement was evaluated by the UPDRS-III. Cognitive changes and motor improvements were compared between PD-MCI and normal cognitive (NC) patients. Logistic regression analyses were performed to explore predictors of post-operative cognitive change.

**Results:**

At the time of surgery, 61.90% of the included PD patients had MCI. Compared with the PD-MCI group, the PD-NC group had a significantly higher proportion of cases with post-operative cognitive decline during follow-up of up to 36 months (mean 17.34 ± 10.61 months), mainly including in global cognitive function, visuospatial/executive function and attention. Covariate-adjusted binary logistic regression analyses showed that pre-operative global cognitive status was an independent variable for post-operative cognitive decline. We also found that pre-operative cognitive specific function could predict its own decline after STN-DBS, except for the naming and orientation domains.

**Conclusion:**

PD-MCI patients are at a lower risk of cognitive decline after STN-DBS compared with PD-NC patients.

## Introduction

Subthalamic nucleus (STN) deep brain stimulation (DBS) has become a well-established treatment for alleviating motor symptoms and reducing the dose of dopaminergic medicine in patients with advanced Parkinson’s disease (PD) (Bronstein et al., 2011; Rughani et al., 2018). But accumulated evidence has shown that subthalamic nucleus deep brain stimulation (STN-DBS) has negative effects on cognitive function in patients with both normal cognitive (NC) and mild cognitive impairment (MCI) (Parsons et al., 2006; Smeding et al., 2011; Kim et al., 2014; Merola et al., 2014; Combs et al., 2015; Odekerken et al., 2015; Cernera et al., 2019; Gruber et al., 2019). MCI represents a transitional cognitive status from normal cognition (NC) to dementia (PDD), and it is common in PD patients, with a prevalence as high as 40% (Hoogland et al., 2017; Gruber et al., 2019; Baiano et al., 2020).

There are relatively few studies on the cognitive outcomes of STN-DBS in PD-MCI patients, and the existing studies have primarily focused on the rate or risk of progression to PDD. These studies have generally shown that PD-MCI patients are at greater risk of developing PDD compared with PD-NC patients (Kim et al., 2014; Merola et al., 2014; Gruber et al., 2019; Park et al., 2020). However, post-operative cognitive decline that does not reach sufficient severity for PDD diagnosis, although does diminish patients’ quality of life, has largely been ignored.

The aim of this retrospective cohort study was to investigate whether the rate of post-operative cognitive decline (not only PDD) was higher in PD-MCI patients compared with PD-NC patients after STN-DBS. We also explored potential baseline parameters that could predict post-operative cognitive decline after STN-DBS.

## Materials and methods

### Patient selection

This was a single center retrospective cohort study. PD patients who underwent STN-DBS between January 2016 and June 2020 were consecutively collected from the database of Beijing Tiantan Hospital (Beijing, China). The inclusion criteria included: (1) idiopathic PD diagnosed according to the UK brain bank criteria; (2) no dementia based on the Mini-Mental State Exam (MMSE) norm for elderly Chinese citizens (MMSE > 20 for individuals with 1–6 years of education and MMSE > 24 for individuals with 7 or more years of education) (Li et al., 2016); (3) bilateral synchronous STN-DBS treatment was performed at Beijing Tiantan Hospital; and (4) complete clinical assessment and follow-up for at least 6 months after STN-DBS. The exclusion criteria included: (1) illiteracy; (2) previous relevant medical history affecting cognitive function (e.g., stroke, brain tumor, hydrocephalus, and brain trauma); (3) serious surgical related complications (e.g., intracerebral hemorrhage); (4) the DBS lead had been revised or replaced; or (5) only online follow-up. This study was approved by the local Institutional Review Board (IRB) (IRB number: KY2020-139-01), all patients provided signed written informed consent.

### Surgical procedures

All operations were performed by the same surgical team, and the surgical process was as described in previous studies in detail (Fan et al., 2020; Yin et al., 2021). Briefly, under local anesthesia, DBS electrodes (model 3,389, Medtronic, Dublin, Ireland, or model L301, Pins Medical, Beijing, China) were implanted with the Leksell micro stereotactic system (Elekta Instrument AB, Stockholm, Sweden). Microelectrode recording and macro stimulation tests were performed for trajectory selection. Then, an implantable pulse generator was implanted into the subclavian region under general anesthesia. Computed tomography scans were performed 1 day after surgery to eliminate complications such as intracranial hemorrhage. The implantable pulse generator was turned on 1 month after the operation. Since then, each patient underwent a regular adjustment of stimulation parameter settings and medication until symptoms were optimally controlled, usually at 6 months after surgery.

### Cognitive and clinical assessment

Cognitive and clinical assessments primarily included cognitive function assessment [i.e., MMSE (Chinese version) and Montreal cognitive assessment (MoCA; Beijing version)], motor function assessment [i.e., Unified Parkinson’s Disease Rating Scale (UPDRS) or Movement Disorder Society (MDS)-UPDRS]. All cognitive and clinical assessments were conducted by the same movement disorder team. Pre-operative assessments were conducted up to 2 weeks before surgery, and regular clinical assessments were conducted at 6 months and 1 year after surgery, and then annually thereafter. All cognitive function assessments were performed in the pre-operative on-medication (med-on) and post-operative on-medication (med-on)/on stimulation (stim-on) states. Motor function assessments were performed as pre-operative: off-medication (med-off) and med-on and post-operative: med-on/stim-on and med-on/stim-off. Med-off status was defined as at least 12 h after the patient withdrew from dopaminergic medications and med-on was defined as 1 h after the patient had taken dopaminergic medications. We also calculated the post- and pre-operative improvement rates of MDS-UPDRS-III in PD patients using the formula described in previous studies (Fan et al., 2020). Levodopa equivalent daily doses (LEDDs) were also recorded.

### Dichotomy of post-operative cognitive decline in individual patients and incidence of post-operative Parkinson’s disease dementia

Pre-operative cognitive status was assessed using the overall MoCA scores. PD-MCI was defined by MoCA scores [≤ 19 for individuals with 1–6 years of education and ≤ 24 for individuals with 7 or more years of education based on the norms for elderly Chinese citizens (Lu et al., 2011)] following MDS diagnostic criteria level I (Litvan et al., 2012). PDD was clinically defined according to MDS diagnostic criteria level I (Dubois et al., 2007). Briefly, MMSE score < 20 for individuals with 1–6 years of education and MMSE < 24 for individuals with 7 or more years of education based on the MMSE norm for elderly Chinese citizens. Based on the changes in MoCA score after STN-DBS, patients were divided into the cognitive-decline and non-cognitive-decline groups. A decrease in MoCA score of > 1.5 standard deviations (SDs) from baseline was defined as individual cognitive decline. Considering that MoCA scores are integers, we rounded 2 points as the cut-off (Lu et al., 2011). And we subtracted 1 point as the cut-off for each specific domain, such as in visuospatial/executive function, naming, attention, language, abstract, delayed recall, and orientation. In addition, the incidence of decline in the MMSE score (2 points as the cut off) during the follow-up was also calculated (Stein et al., 2012). In addition, the percentage of decline in the MMSE score (2 points as the cut off) during the follow-up was also calculated (Stein et al., 2012).

### Potential predictors of post-operative cognitive decline

We collected baseline information from PD patients as potential predictors of cognitive decline, including gender, age at surgery, age of onset, disease duration, education, Hoehn–Yahr stage (med-off), MDS-UPDRS-III (med-off), levodopa responsiveness, symptom onset side, LEDD and pre-operative global cognitive status (PD-NC and PD-MCI, defined by pre-operative overall MoCA score). This study used 50-years-old as the cut-off value for age of onset, and PD patients were divided into early-onset PD (< 50) and late-onset PD (≥ 50) (Schrag and Schott, 2006). Hoehn–Yahr stage was only collected in the med-off state. Levodopa responsiveness was calculated as (UPDRS-III [med-off]–UPDRS-III [med-on])÷UPDRS-III [med-off] × 100%. It should be noted that because some patients were assessed with UPDRS and others with MDS-UPDRS (the scale used for each patient was consistent during follow-up), UPDRS-III scores were uniformly converted to MDS-UPDRS-III using the previously reported method (Goetz et al., 2012).

### Statistical analysis

Descriptive statistics were used to describe baseline and follow-up demographic data. Continuous variables are described as mean (± SD), while categorical variables are expressed as percentages. Intergroup comparisons of demographic data between patients were performed by applying either the independent *t*-test for continuous variables or the chi-square test for binary variables.

First, in order to investigated whether there were differences in cognitive decline after STN-DBS between the two groups, the two-way repeated-measures ANOVA were performed, with group (PD-MCI and PD-NC) as the between-group factor and time (before and after DBS) as the within-group factor, followed by Bonferroni’s *post-hoc* tests. Proportions of post-operative cognitive decline at last follow-up as well as the rate of cognitive decline at each follow-up time point were compared between groups using the Chi-square or Fisher’s exact test. In addition, incidence of post-operative PDD were also compared between groups using the Chi-square test. Second, we evaluated baseline variables that could predict cognitive decline using univariate logistic regression models. Because no consistent predictors have been identified in previous studies, we included all available parameters as an exploratory analysis, the stepwise forward approach was used to input these measurements as independent variables into the multivariable logistic regression model. Then, we also used the logistic regression model to explore possible baseline cognitive variables that could predict decline in cognitive specific domains following STN-DBS. Two tailed *p*-values < 0.05 were considered significant. Reported *p*-values were corrected for multiple comparisons in the domain analysis using the Bonferroni test (initial α = 0.05). All statistical analyses were performed using The R Project for Statistical Computing (version 3.6.1).^1^

## Results

### Baseline characteristics of the Parkinson’s disease-mild cognitive impairment and Parkinson’s disease-normal cognition groups

**FIGURE 1:**
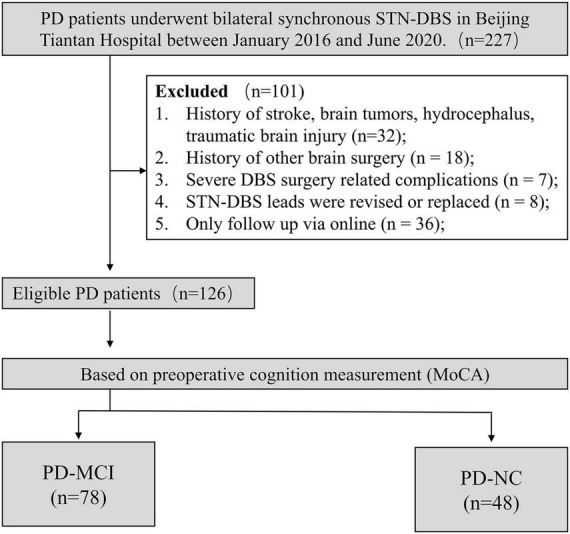
Flow chart of participant identification. PD-MCI, Parkinson’s disease with mild cognitive impairment; PD-NC, Parkinson’s disease with normal cognition; MoCA, Montreal Cognitive Assessment; MMSE, Mini-Mental State Examination.

The detailed workflow is shown in Figure 1. A total of 126 PD patients were ultimately included in this study. Among the included patients, 61.90% had MCI status at the time of surgery. As shown in Table 1, the PD-MCI group had lower MMSE (PD-MCI: 26.73 ± 2.12; PD-NC: 28.15 ± 1.90) and MoCA (PD-MCI: 19.33 ± 3.39; PD-NC: 25.19 ± 1.79) scores, decreased scores in all the specific cognitive domain of MoCA, and fewer years of education. The two groups did not differ significantly in any other baseline characteristics.

**TABLE 1:** Baseline characteristics and clinical information of PD patients with MCI and NC.

Variables	PD-MCI (*n* = 78)	PD-NC (*n* = 48)	*P*-value
Gender/female (%)^a^	37 (47.44%)	17 (35.42%)	0.186
Age of surgery (years)	62.88 ± 8.45	61.69 ± 8.58	0.444
Age of onset/late onset (%)^a^	52 (66.67%)	33 (68.75%)	0.808
Duration of disease (years)	9.72 ± 4.28	8.92 ± 3.49	0.277
Education (years)	9.96 ± 2.96	11.92 ± 3.00	<0.001*
Hoehn Yahr stage (med off)	2.96 ± 0.57	3.01 ± 0.63	0.650
LEDD (mg)	711.82 ± 368.24	749.14 ± 409.51	0.598
MDS-UPDRS-III (med off)	55.11 ± 19.06	58.03 ± 21.52	0.428
Levodopa responsiveness (%)	50.35 ± 19.26	52.16 ± 17.12	0.652
Symptom onset side (left/right/bilateral)^a^	32/39/2	19/21/5	0.169
MMSE score	26.73 ± 2.12	28.15 ± 1.90	<0.001*
MoCA score	19.21 ± 3.81	25.19 ± 1.79	<0.001*
MoCA specific domains score			
Visuospatial/executive function score	2.37 ± 1.43	3.96 ± 1.01	<0.001*
Naming score	2.53 ± 0.83	2.90 ± 0.31	0.004*
Attention score	4.94 ± 1.30	5.65 ± 0.56	<0.001*
Language score	1.90 ± 0.85	2.58 ± 0.61	<0.001*
Abstract score	0.97 ± 0.84	1.56 ± 0.54	<0.001*
Delayed recall score	1.08 ± 1.09	2.71 ± 1.32	0.025*
Orientation score	5.42 ± 1.19	5.83 ± 0.48	<0.001*

^a^Chi-square test; unindicated comparisons were conducted using the independent t-test; Significant results are marked with*.

PD-MCI, Parkinson’s disease with mild cognitive impairment; PD-NC, Parkinson’s disease with normal cognition; LEDD, Levodopa equivalent dose; MDS-UPDRS-IIIPDRSi-off), Movement Disorder Society-Unified Parkinson’s Disease Rating Scale, Part III (med-off); MoCA, Montreal Cognitive Assessment; MMSE, Mini-Mental State Examination (off medication).

### Post-operative decline in cognitive test scores between groups

**FIGURE 2:**
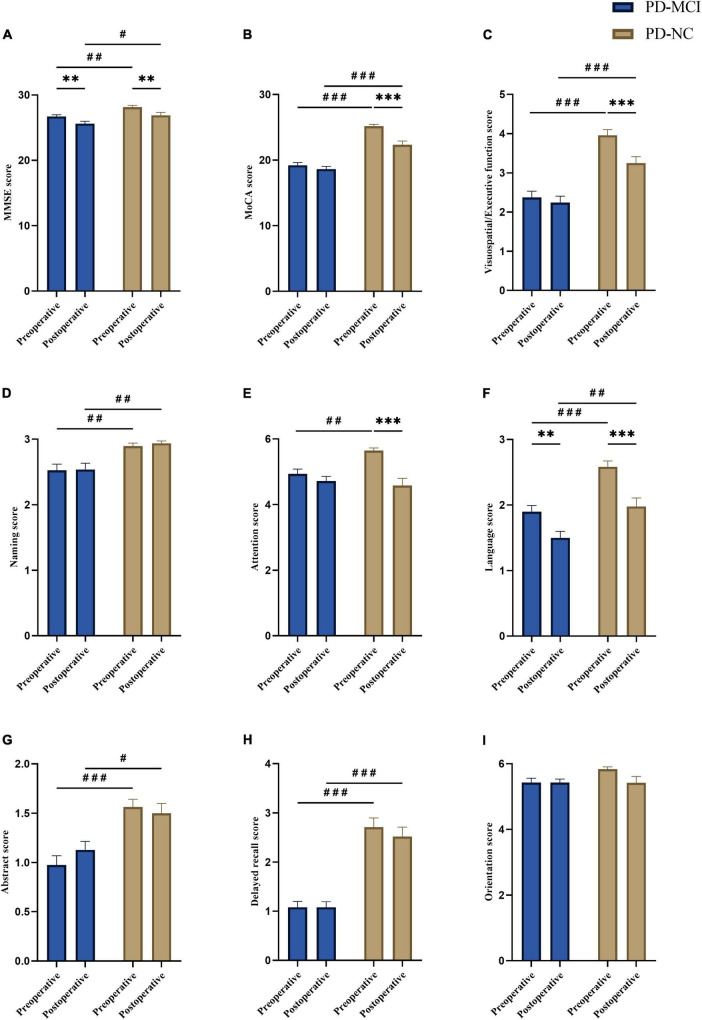
Comparison of pre- and post-operative cognitive scores between groups. Compare to pre-operative, **(A)** overall MMSE scores (PD-MCI: **p = 0.002; PD-NC: **p = 0.006) and language scores (PD-MCI: **p = 0.001; PD-NC: ***p < 0.001) showed decline in PD-MCI and PD-NC groups. **(B)** Overall MoCA scores (***p < 0.001), **(C)** visuospatial/executive function (***p < 0.001) and **(E)** attention (***p < 0.001) showed significant post-operative decline in PD-NC group. Compare to PD-NC group, PD-MCI group showed lower score in **(A)** overall MMSE (pre-operative: ^##^*p* = 0.009; post-operative: ^#^*p* = 0.02), **(B)** overall MoCA (pre-operative: ^###^*p* < 0.001; post-operative: ^###^*p* < 0.001), **(C)** visuospatial/executive function (pre-operative: ^###^*p* < 0.001; post-operative: ^###^*p* < 0.001), **(D)** naming (pre-operative: ^##^*p* = 0.007; post-operative: ^##^*p* = 0.003), **(F)** language (pre-operative: ^###^*p* < 0.001; post-operative: ^##^*p* = 0.004), **(G)** abstract (pre-operative: ^###^*p* < 0.001; post-operative: ^#^*p* = 0.013), and **(H)** delayed recall (pre-operative: ^###^*p* < 0.001; post-operative: ^###^*p* < 0.001) in both pre- and post-operative, and **(E)** attention (^##^*p* = 0.003) in pre-operative. No significant difference was found in orientation (between pre- and postoperative within-group, or between-groups) **(I)**. The data were analyzed by two-way repeated-measures ANOVA with group (PD-MCI and PD-NC) as the between group factor and time (before and after DBS) as the within group factor, followed by Bonferroni’s *post-hoc* tests. All graphs are depicted with bars for standard errors of the mean (SEM) and *p*-values (two-tailed) with the following significance level: **p < 0.01; ***p < 0.001 (within-group comparison); ^#^*p* < 0.05; ^##^*p* < 0.01; ^###^*p* < 0.001 (between-group comparison). PD-MCI, Parkinson’s disease with mild cognitive impairment; PD-NC, Parkinson’s disease with normal cognition; MoCA, Montreal Cognitive Assessment; MMSE, Mini-Mental State Examination.

We have presented the results of two-way repeated-measures ANOVA in Figure 2 and Supplementary Table 1. Mean cognitive test scores significantly decreased following STN-DBS on indices of MMSE (*F* = 20.35, *p* < 0.001) and MoCA (*F* = 36.12, *p* < 0.001), as well as MoCA specific domains, including visuospatial/executive function (*F* = 12.72, *p* < 0.001), attention (*F* = 26.25, *p* < 0.001) and language (*F* = 29.77, *p* < 0.001). *Post hoc* analyses revealed that, for MMSE (Figure 2A; PD-MCI: *t* = 3.45, *p* = 0.002; PD-NC: *t* = 3.03, *p* = 0.006) and language (Figure 2F; PD-MCI: *t* = 3.51, *p* = 0.001; PD-NC: *t* = 4.18, *p* < 0.001), both groups demonstrated significant post-operative decline. While for MoCA (Figure 2B; PD-MCI: *t* = 1.64, *p* = 0.21; PD-NC: *t* = 6.35, *p* < 0.001), visuospatial/executive function (Figure 2C; PD-MCI: *t* = 0.89, *p* = 0.75; PD-NC: *t* = 3.84, *p* < 0.001) as well as attention (Figure 2E; PD-MCI: *t* = 1.41, *p* = 0.32; PD-NC: *t* = 5.40, *p* < 0.001), the *post hoc* analysis showed significant post-operative decline compared pre-operative scores at PD-NC group only. There was also a significant Group X Time interaction effect demonstrating greater post-operative cognitive decline in patients with PD-NC compared to PD-MCI on MoCA (*F* = 15.91, *p* < 0.001) and MoCA specific domains, including visuospatial/executive function (*F* = 6.12, *p* = 0.015) and attention (*F* = 11.42, *p* < 0.001) (Supplementary Table 1). In addition, post-operative cognitive scores except attention were significantly higher in PD-NC group than in PD-MCI group (Figure 2).

Further, patients in the PD-NC group were divided into two sub-groups [declined group (*n* = 27) and non-declined group (*n* = 21)] according to whether they had experienced post-operative cognitive (MoCA) decline, and the independent *t*-test was performed to compare pre-operative MoCA scores between the two PD-NC sub-groups. Results showed no statistical difference of pre-operative MoCA scores between the declined group (25.30 ± 2.22) and non-declined group (25.05 ± 1.07) (*p* = 0.64).

### Rates of post-operative cognitive decline between groups

The comparison of post-operative cognitive decline rates at last follow-up between PD-MCI and PD-NC groups are displayed in Table 2. Mean duration of follow-up seemed to be longer in the PD-NC group than in the PD-MCI group, but group differences were not statistically significant (*p* = 0.324). At last follow-up, significant decline rate of MMSE (PD-MCI: 29.49%; PD-NC: 22.92%), MoCA (PD-MCI: 30.77%; PD-NC: 56.25%), and MoCA specific domains, including visuospatial/executive function (PD-MCI: 33.33%; PD-NC: 56.25%), attention (PD-MCI: 43.59%; PD-NC: 62.50%) and language (PD-MCI: 42.31%; PD-NC: 47.92%) were observed in both groups after STN-DBS. And a higher proportion of post-operative PDD cases were also found in the PD-MCI group compared with PD-NC group (19.20% vs. 6.25%, *p* = 0.043) (Table 2). There was a higher percentage of patients with decreased MoCA scores in the PD-NC group than in the PD-MCI group (*p* = 0.005). Furthermore, the PD-NC group showed a higher proportion of declines in specific MoCA domains including visuospatial/executive function, attention and delayed recall. Both groups had an excellent rate of improvement in MDS-UPDRS-III after surgery, with no significant difference found between the two groups (*p* = 0.081).

**TABLE 2:** Percentages of cognitive decline and rates of improved MDS-UPDRS-III outcomes at the last follow-up between the PD-MCI and PD-NC groups.

Variables	PD-MCI (*n* = 78)	PD-NC (*n* = 48)	*P*-value
Follow-up (months)^a^	16.08 ± 10.48	18.78 ± 11.43	0.324
Decline of MMSE	23 (29.49%)	11(22.92%)	0.420
Decline of MoCA	24 (30.77%)	27 (56.25%)	0.005*
Decline of MoCA specific domains			
Visuospatial/Executive function	26 (33.33%)	27 (56.25%)	0.011*
Naming	9 (11.54%)	3 (6.25%)	0.326
Attention	34 (43.59%)	30 (62.50%)	0.039*
Language	33 (42.31%)	23 (47.92%)	0.538
Abstract	19 (24.36%)	10 (20.83%)	0.648
Delayed recall	12 (15.38%)	20 (41.67%)	<0.001*
Orientation	21 (26.92%)	8 (16.67%)	0.184
PDD	15 (19.20%)	3 (6.25%)	0.043*
Improvement rate of MDS-UPDRS-III (%)^a^	45.37 ± 24.16	54.24 ± 30.36	0.081

^a^Independent t-test; unindicated comparisons were conducted using Chi-square tests or Fisher’s exact test; Significant results are marked with*.

PD-MCI, Parkinson’s disease with mild cognitive impairment; PD-NC, Parkinson’s disease with normal cognition; MoCA, Montreal Cognitive Assessment; MMSE, Mini-Mental State Examination; MDS-UPDRS-IIIP Movement Disorder Society-Unified Parkinson’s Disease Rating Scale, Part III, PDD, Parkinson’s disease dementia.

To eliminate the influence of follow-up duration, we further stratified results by follow-up time (6, 12, 24, and 36 months). The results are displayed in Table 3. At 24 and 36 months following STN-DBS, the decline rates of MoCA (24 months, PD-MCI: 31.25%, PD-NC: 77.78%, *p* = 0.041; 36 months, PD-MCI: 25.00%, PD-NC: 75.00%, *p* = 0.039) and visuospatial/executive function (24 months, PD-MCI: 25.00%, PD-NC: 77.78%, *p* = 0.017; 36 months, PD-MCI: 25.00%, PD-NC: 75.00%, *p* = 0.039) were significantly higher in the PD-NC group compared to that in the PD-MCI group. We also found attention remained significantly higher in the PD-NC (56.25%) compared to PD-MCI group (22.58%) at 12 months after surgery (*p* = 0.021). Although the decline rates of delayed recall (memory) in the PD-NC group were higher than those in the PD-MCI group at each follow-up timepoint, there were no significant differences between the two groups (Table 3).

**TABLE 3:** Percentages of cognitive decline at each follow-up timepoint between the PD-MCI and PD-NC groups.

Decline rates	6 months			12 months			24 months			36 months		
				
	PD-MCI (*n* = 19)	PD-NC (*n* = 11)	*P*-value	PD-MCI (*n* = 31)	PD-NC (*n* = 16)	*P*-value	PD-MCI (*n* = 16)	PD-NC (*n* = 9)	*P*-value	PD-MCI (*n* = 12)	PD-NC (*n* = 12)	*P*-value
MMSE	6 (31.58%)	1 (9.10%)	0.215	6 (19.35%)	4 (25.00%)	0.943	7 (43.75%)	3 (33.33%)	0.691	4 (33.33%)	3 (25.00%)	>0.999
MoCA	8 (42.11%)	5 (45.45%)	>0.999	8 (25.81%)	6 (37.50%)	0.621	5 (31.25%)	7 (77.78%)	0.041*	3 (25.00%)	9 (75.00%)	0.039*
MoCA specific domains												
Visuospatial/executive	7 (36.84%)	4 (36.36%)	>0.999	12 (38.71%)	7 (43.75%)	0.739	4 (25.00%)	7 (77.78%)	0.017*	3 (25.00%)	9 (75.00%)	0.039*
Naming	2 (10.53%)	0 (0)	0.529	2 (6.45%)	1 (6.25%)	>0.999	2 (12.50%)	0 (0%)	0.529	3 (25.00%)	2 (16.67%)	>0.999
Attention	9 (47.37%)	4 (36.36%)	0.708	7 (22.58%)	9 (56.25%)	0.021*	7 (43.75%)	6 (66.67%)	0.411	11 (91.67%)	11 (91.67%)	>0.999
Language	10 (52.63%)	5 (45.45%)	>0.999	9 (29.03%)	3 (18.75%)	0.680	4 (25.00%)	4 (44.44%)	0.394	10 (83.33%)	11 (91.67%)	>0.999
Abstract	5 (26.32%)	1 (9.10%)	0.372	8 (25.81%)	6 (37.50%)	0.621	3 (18.75%)	1 (11.11%)	>0.999	3 (25.00%)	2 (16.67%)	>0.999
Delayed recall	4 (21.05%)	5 (45.45%)	0.225	4 (12.90%)	6 (37.50%)	0.115	2 (12.50%)	5 (55.56%)	0.058	2 (16.67%)	4 (33.33%)	0.640
Orientation	5 (26.32%)	1 (9.10%)	0.372	2 (6.45%)	1 (6.25%)	>0.999	9 (56.25%)	3 (33.33%)	0.411	5 (41.67%)	3 (25.00%)	0.667

Comparisons were conducted using Chi-square tests or Fisher’s exact test; Significant comparisons are marked with *.

PD-MCI, Parkinson’s disease with mild cognitive impairment; PD-NC, Parkinson’s disease with normal cognition; MoCA, Montreal Cognitive Assessment; MMSE, Mini-Mental State Examination.

### Pre-operative predictors of post-operative cognitive decline

Two potential baseline predictors of cognitive decline were entered into multivariate logistic regression models: pre-operative global cognitive status (PD-NC and PD-MCI, defined by pre-operative MoCA score) [odds ratio (OR): 2.89, 95% confidence interval (CI): 1.37–6.10, *p* = 0.005] and UPDRS-III (med-off) (OR: 0.98, 95%CI: 0.97–1.00, *p* = 0.084; Table 4). The multivariate logistic regression model was adjusted twice: (1) for age and gender (adjust I model); and (2) for age, gender, age of onset, education, Hoehn–Yahr stage (med-off), and symptom onset side (adjust II model). After adjusting other factors, the pattern remained unchanged, and the stepwise forward regression model showed that pre-operative global cognitive status (PD-NC) at baseline was an independent variable (adjusted OR: 2.961, 95%CI: 1.191–7.357, *p* = 0.020) for post-operative cognitive decline (Table 5). The multivariate logistic regression model showed that at baseline, PD-MCI patients had a significantly lower probability of cognitive decline after STN-DBS than PD-NC patients.

**TABLE 4:** Impact of pre-operative cognitive status, clinical and demographic data on post-operative cognitive decline (univariate logistic regression models).

Variables	OR	95% CI	*P*-value
Gender/female (%)	0.89	(0.43, 1.83)	0.753
Age of surgery (years)	1.00	(0.96, 1.04)	0.895
Age of onset/late onset	0.94	(0.44, 2.01)	0.875
Duration of disease (years)	1.02	(0.93, 1.11)	0.715
Education (years)	1.00	(0.89, 1.13)	0.952
Hoehn Yahr stage (med off)	0.78	(0.42, 1.45)	0.436
LEDD (mg)	1.00	(1.00, 1.00)	0.112
MDS-UPDRS-III (med off)	0.98	(0.97, 1.00)	0.084*
Levodopa responsiveness (%)	1.18	(0.17, 8.27)	0.870
Symptom onset side/right	0.95	(0.45, 2.04)	0.900
Symptom onset side/bilateral	1.90	(0.39, 9.41)	0.430
Pre-operative cognitive status/PD-NC	2.89	(1.37, 6.10)	0.005*

Significant comparisons are marked with *.

PD-NC, Parkinson’s disease with normal cognition; LEDD, Levodopa equivalent dose; MDS-UPDRS-III (med-off), Movement Disorder Society-Unified Parkinson’s Disease Rating Scale, Part ty(med-off); MoCA, Montreal Cognitive Assessment; MMSE, Mini-Mental State Examination (off medication). OR, odds ratio; CI, confidence interval.

**TABLE 5:** Impact of pre-operative cognitive status (PD-NC) and MDS-UPDRS-III (med-off) on post-operative cognitive decline (multivariate logistic regression models).

Variables	Non-adjusted			Adjust -I			Adjust -II		
			
	OR	95% CI	*P*-value	OR	95% CI	*P*-value	OR	95% CI	*P*-value
Pre-operative cognitive status/PD-NC	2.832	(1.332, 6.020)	0.007*	2.968	(1.357, 6.492)	0.006*	2.961	(1.191, 7.357)	0.020*
MDS-UPDRS-III (med off)	1.015	(0.996, 1.035)	0.113	1.019	(0.999, 1.040)	0.060	1.019	(0.994, 1.045)	0.136

Significant comparisons are marked with *; non-adjusted model adjusted for: none; adjusted model I adjusted for: age and gender; adjusted model II adjusted for: age, gender, age of onset, education, Hoehn–Yahr stage (med-off), and symptom onset side.

PD-NC, Parkinson’s disease with normal cognition; LEDD, Levodopa equivalent dose; MDS-UPDRS-III (med-off), Movement Disorder Society-Unified Parkinson’s Disease Rating Scale, Part III (off medication); OR: odds ratio; CI, confidence interval.

**FIGURE 3:**
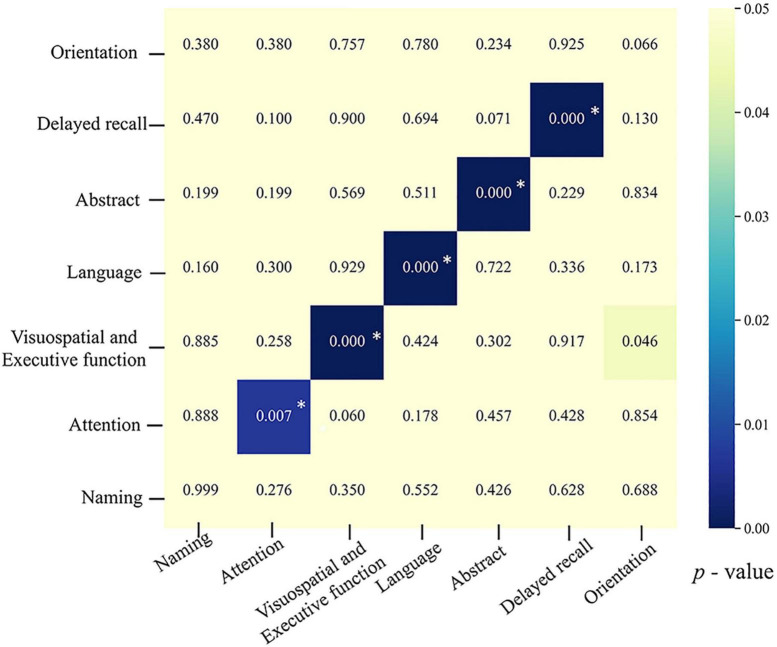
Heatmap showing outcomes of predictors of post-operative decline in specific MoCA domains. The x-axis shows post-operative decline in seven different MoCA domains based on which cognitive-decline dichotomy was made. The y-axis lists seven potential predictors. The color of the blocks reflects the *p*-value from comparisons of each potential predictor between the cognitive-decline group and the non-decline group, with darker color indicating smaller *p*-values. After Bonferroni correction, *p* < 0.008 (0.05/7) was considered significant. Blocks with *indicate factors that remained statistically significant in the multivariate regression model.

Furthermore, we established a logistic regression model to explore predictors of cognitive decline in specific MoCA domains. These data indicated that pre-operative cognitive specific function could predict its own decline after STN-DBS, except for the naming and orientation domains (Figure 3). All results were corrected for multiple comparisons using the Bonferroni test (initial α = 0.05).

## Discussion

In this study, first, we compared the pre- and post-operative cognitive scores as well as proportions of post-operative cognitive decline of PD patients with different pre-operative cognitive status (including PD-NC and PD-MCI, defined by pre-operative overall MoCA score). Results of cognitive scores showed that global cognition (MoCA and MMSE), visuospatial/executive function, attention, and language scores significantly decreased in both groups following STN-DBS, and greater post-operative cognitive decline in patients with PD-NC compared to PD-MCI on MoCA, visuospatial/executive function and attention scores. The percentage of patients with post-operative cognitive decline at last follow-up was higher in the PD-NC group compared with the PD-MCI group, including overall MoCA, visuospatial/executive function, attention, and delayed recall (memory). However, when stratifying by duration of follow-up, results showed that decline rates of overall MoCA (at 24 and 36 months after surgery) and visuospatial/executive function (at 24 and 36 months after surgery), as well as attention (at 12 months after surgery) remained significantly higher in the PD-NC group compared to that in the PD-MCI group. Then, potential predictors of post-operative cognitive decline were also explored, which revealed that patients with pre-operative NC status had a significantly higher probability of cognitive decline after STN-DBS than patients with MCI status.

Our data showed that with an average disease duration of 9.41 years, the prevalence of MCI was 61.90% at the time of surgery, which was consistent with previously studies. It was reported that prevalence of MCI was 63.1% among 103 non-demented PD patients with a disease duration of 10.6 years (Kim et al., 2014), similarly, another study reported a 69.08% rate of MCI among 304 PD patients with an average disease duration of 13 years (Park et al., 2020). Notably, the prevalence of MCI was high in our cohort compared with a meta-analysis of 7,053 non-surgery PD patients that had a pooled prevalence of PD-MCI of 40% (Baiano et al., 2020). The differences between their results and those presented herein may be attributable to differences in the patient cohort. In our study, patients mostly had advanced PD, longer disease duration, increased disease severity, and needed surgery. Overall, MCI is highly common among PD patients undergoing DBS surgery, so more attention should be paid to this patient population.

The percentage of patients with post-operative global cognitive (MoCA) decline among all PD patients at the last follow-up (mean 17.52 months) was 40.48%, which was very close to the rate reported by prior work (39.3%), where patients were not classified as PD-NC or PD-MCI at baseline and cognitive decline after 12 months was defined as a significant deterioration of three or more cognitive tests according to the reliable change index (Odekerken et al., 2015). Our findings are also similar with another study, which showed that at the individual level, compared with the control group, 36% of patients in the STN-DBS group showed cognitive decline at 12-month follow-up (Smeding et al., 2011).

In line with prior studies (York et al., 2008; Zangaglia et al., 2009; Kim et al., 2014; Park et al., 2020), this study showed that post-operative MMSE scores of the two groups were significantly lower than pre-operative scores, and significant post-operative decline rate of MMSE scores was also found in both groups, but no statistically significant differences were noticed between groups. In addition, we found that patients with PD-MCI (19.20%) progressed to PDD in higher proportions than patients with PD-NC (6.25%) at the last follow-up, which has been demonstrated by previous studies (Merola et al., 2014; Gruber et al., 2019). It was reported that patients with MCI (46.4%) had a markedly higher prevalence of developing PDD following STN-DBS compared with NC patients (22.2%) after 6.3 years follow-up (Gruber et al., 2019). The post-operative median time for PD-MCI patients to develop PDD was 6.03 years compared with 11.08 years for those with PD-NC (Merola et al., 2014). There have also been several predictive studies that have reported an increased risk of PDD for MCI subjects compared with NC individuals following STN-DBS (Kim et al., 2014; Park et al., 2020).

However, our study showed that the proportion of patients with global cognitive (MoCA) decline in the PD-NC group (56.25%) was higher than in the PD-MCI group (30.77%). Meanwhile, multivariate logistic regression results also confirmed that the risk of cognitive decline after STN-DBS in the PD-NC group was increased compared with the PD-MCI group. The difference between our data and previous studies may be due to the following reasons: (1) we primarily focused on post-operative cognitive decline, not only cases that progressed to PDD; and (2) we used the MoCA scale for cognitive assessment, while other studies have used the MDRS (Gruber et al., 2019), MMSE (Kim et al., 2014; Park et al., 2020), and other comprehensive scales (Merola et al., 2014).

For the decline of specific domains in MoCA, the findings were generally consistent across both methodologies (comparison of change in cognitive scores and analysis the of cognitive decline rate). First, our results demonstrate that both groups showed a significant post-operative decline in terms of visuospatial/executive function, attention and language, which is consistent with these previous studies (York et al., 2008; Okun et al., 2009; Fasano et al., 2010; Merola et al., 2011; Smeding et al., 2011; Georgiev et al., 2021). Second, patients with PD-NC show more declines on visuospatial/executive function and attention, also memory. Of these, visuospatial/executive function and attention remained significantly after considering the influence duration of follow-up.

The findings above can be explained as follows. The STN can be subdivided into three functional zones, including the dorsolateral region connects with motor pathways, the central region connects with associative pathways, and the ventromedial region connects with limbic pathways. Notably, the associative pathway receives mainly inputs from the frontal cortex, and plays an essential role in regulating cognitive function (Karachi et al., 2005; Temel et al., 2005). And substantial overlap between its functional zones as its compact structure (Balaz et al., 2011; Alkemade and Forstmann, 2014). Language, especially verbal fluency relies more on prefrontal and anterior cingulate, premotor cortex and ventral part of the caudate (Cilia et al., 2007; Mikos et al., 2011). In addition to the associative pathway, evidence indicates that there is also a hyper-direct pathway between the STN and frontal cortex through low-frequency interactions that support executive function (Zavala et al., 2014; Kelley et al., 2018). Attention and executive function are inextricably linked. Some even suggested that attention is part of an executive function (Kehagia et al., 2010). Attention is associated with the frontal and parietal cortex (called frontal-parietal network) (Buschman and Miller, 2007). Consequently, cognitive function including executive function, language and attention will likely be influenced by the STN-DBS, as the disruption of frontal-striatal connectivity, even though clinical DBS was performed on motor sites in the dorsolateral STN. As PD-NC show more declines in visuospatial/executive and attention, it is likely that PD-MCI patients had impairments in these cognitive domains at baseline (Aarsland et al., 2010; Kalbe et al., 2016; Nie et al., 2019), and their post-operative deterioration was not as apparent as in the PD-NC group.

This study also found that the pre-operative status of specific MoCA domains, except for the naming domain, could predict their own decline after STN-DBS. Our findings are generally consistent with prior studies (Odekerken et al., 2015; Hogue et al., 2018). This finding might be explained by the low rate of patients with a post-operative decrease in naming (PD-MCI: 11.54%, PD-NC: 6.25%, total: 9.52%).

There were a few limitations to this study. First, this was a single center retrospective study with its inherent defects, including being subject to bias, even though patients were enrolled consecutively. Our findings need to be confirmed by further prospective longitudinal studies. Second, the present study used only MoCA to determine cognitive status. Nevertheless, comprehensive neuropsychological assessments are not always available or feasible in clinical practice. Despite the limitation, our findings can be considered to provide a reference for clinicians, which reminds us that clinicians need to focus not only on the patients with pre-operative impaired cognitive function but also on the patients with normal cognitive status in future clinical trials, a comprehensive neuropsychological battery test should be employed to assess the cognitive function of PD patients. Actually, a subsequent ongoing study with a larger patient cohort and a more detailed comprehensive neuropsychological battery according to MDS diagnostic criteria level II (Dubois et al., 2007; Litvan et al., 2012) is under way at our center, which will allow for more detailed and accurate analyses of cognitive function. Third, we did not set up a control group of medically treated patients, making it difficult to rule out the impact of disease progression on cognitive decline following STN-DBS.

## Conclusion

In conclusion, our data indicated that compared with PD-NC, PD-MCI patients had lower risk of cognitive decline after STN-DBS, and their rate of motor improvement was similar, although PD-MCI patients progressed to PDD in higher proportions than PD-NC individuals.

## Data availability statement

The raw data supporting the conclusions of this article will be made available by the authors, without undue reservation.

## Ethics statement

The studies involving human participants were reviewed and approved by the Local Institutional Review Board (IRB) (IRB number: KY2020-139-01). The patients/participants provided their written informed consent to participate in this study.

## Author contributions

HX, YB, QZ, YJ, and JZ: conceptualization and resources. HX, YB, and QZ: data curation and investigation. HX and YB: formal analysis, validation, and writing—original draft. JZ: funding acquisition and project administration. HX, YB, YJ, and JZ: methodology. YB, YJ, and JZ: writing—review, editing, and supervision. All authors contributed to the article and approved the submitted version.

## Footnotes

1
https://www.rstudio.com/
