# Supplementary material for: Parkinson’s disease with mild cognitive impairment may has a lower risk of cognitive decline after subthalamic nucleus deep brain stimulation: A retrospective cohort study
Source: Front Hum Neurosci. 2022 Sep 6;16:943472. doi: 10.3389/fnhum.2022.943472 (PMC9486063; doi:10.3389/fnhum.2022.943472)
Supplement: Supplementary file 1 [file Table_1.docx]

**Supplementary Table 1.**

Pre- and post-operative cognitive test scores at the last follow-up between the PD-MCI and PD-NC groups.

| **Variables** | **PD-MCI**  **(n = 78)** | | **PD-NC**  **(n = 48)** | | **Interaction effect** |
| --- | --- | --- | --- | --- | --- |
|  | **Pre-operative** | **Post-operative** | **Pre-operative** | **Post-operative** | ***p-*value** |
| **MMSE score** | 26.73 ± 2.12 | 25.62 ± 3.25 | 28.15 ± 1.90 | 26.90 ± 3.12 | 0.798 |
| **MoCA score** | 19.21±3.81 | 18.63 ±3.78 | 25.19 ± 1.79 | 22.33 ± 4.05 | < 0.001^*^ |
| **MoCA specific domains** |  |  |  |  |  |
| **Visuospatial/**  **Executive score** | 2.37 ± 1.43 | 2.24 ± 1.43 | 3.96 ± 1.01 | 3.25 ± 1.14 | 0.015^*^ |
| **Naming score** | 2.53 ± 0.83 | 2.54 ± 0.85 | 2.90 ± 0.31 | 2.94 ± 0.24 | 0.779 |
| **Attention score** | 4.94 ± 1.30 | 4.72 ±1.23 | 5.65 ± 0.56 | 4.56 ± 1.63 | < 0.001^*^ |
| **Language score** | 1.90 ± 0.85 | 1.50 ± 0.88 | 2.58 ± 0.61 | 1.98 ± 0.89 | 0.262 |
| **Abstract score** | 0.97 ± 0.84 | 1.13 ± 0.76 | 1.56 ± 0.54 | 1.50 ± 0.68 | 0.211 |
| **Delayed recall score** | 1.08 ± 1.09 | 1.08 ± 1.03 | 2.71 ± 1.32 | 2.52 ± 1.32 | 0.412 |
| **Orientation score** | 5.42 ± 1.19 | 5.42 ± 0.97 | 5.83 ± 0.48 | 5.42 ± 1.37 | 0.086 |

Comparisons were conducted using the two-way repeated-measures ANOVA with group (PD-MCI and PD-NC) as the between-group factor and time (pre- and post-operative) as the within-group factor; Significant interaction effect are marked with *.

Abbreviations: PD-MCI, Parkinson's disease with mild cognitive impairment; PD-NC, Parkinson's disease with normal cognition; MoCA, Montreal Cognitive Assessment; MMSE, Mini-Mental State Examination.
